# Challenges in Diagnosing Central Adrenal Insufficiency in Children: Cortisol‐Stimulating Tests are Safe and Often Required

**DOI:** 10.1111/cen.70164

**Published:** 2026-05-17

**Authors:** Mariana Peduti Halah, Ana Carolina Maia Teodózio, Davi Casale Aragon, Paula Conde Lamparelli Elias, Margaret de Castro, Ayrton Custodio Moreira, Sonir Rauber Antonini

**Affiliations:** ^1^ Division of Pediatric Endocrinology—Department of Pediatrics, Ribeirão Preto Medical School University of São Paulo Brazil; ^2^ Statistician—Department of Pediatrics, Ribeirão Preto Medical School University of São Paulo Brazil; ^3^ Division of Endocrinology and Metabolism—Department of Internal Medicine, Ribeirão Preto Medical School University of São Paulo Brazil

**Keywords:** adrenal gland diseases, adrenal insufficiency, hypopituitarism, hypothalamic‐pituitary‐adrenal axis, pituitary gland, pituitary‐adrenal function tests, pituitary‐adrenal system

## Abstract

**Introduction:**

The accuracy and safety of cortisol‐stimulating tests (CSTs) for assessing hypothalamic–pituitary–adrenal (HPA) axis integrity, including the diagnosis of central adrenal insufficiency (CAI), in children remain uncertain. Although these tests can simultaneously evaluate cortisol and growth hormone secretion, the present study focuses exclusively on cortisol measurements.

**Methods:**

We evaluated the diagnostic accuracy and safety profile of CST in pediatric patients submitted to insulin tolerance test (ITT) or glucagon‐stimulation test (GST) in a tertiary center for 15 years in this retrospective cohort study. According to cortisol (F; nmol/L), we classified patients as having CAI (Fbasal < 138 or Fpeak < 414), indeterminate (Fbasal = 138−358.7 or Fpeak = 414−496.6), and normal (Fbasal > 358.7 or Fpeak > 496.6). Cutoffs were selected based on the literature and on our University Hospital protocol. Plasma cortisol was measured using a consistent radioimmunoassay (RIA) following extraction, with duplicate analyses. The assay had a detection limit of 33.11 nmol/L, with intra‐ and inter‐assay coefficients of variation of 5% and 10.5%, respectively. We evaluated the sensitivity, specificity, likelihood ratios (LR), and clinicopathological variables associated with Fbasal and Fpeak.

**Results:**

Among 904 patients (60.1% males; median age 10.1 years [0.1−17.1]), CAI was confirmed in 79 (8.7%). Mild hypoglycemia (ITT), and nausea and vomiting (GST) occurred in 46% and 17.5%, respectively. No serious adverse events occurred. Fbasal classified 58% of patients as indeterminate. A Fbasal cutoff of 96.6 had a specificity of 99% and LR+ of 7.6, but low sensitivity (5.3%). In GST, a Fpeak of 303.5 had weak diagnostic accuracy (LR+ of 2.8) and low sensitivity (17%). Concomitant central hypothyroidism and a Fbasal < 154.5 were associated with lower Fpeak, and pituitary stalk interruption was associated with lower Fbasal and Fpeak.

**Conclusions:**

Fbasal did not present good diagnostic accuracy. However, Fbasal < 96.6 nmol/L predicted lower Fpeak. Therefore, many patients require CST to confirm or exclude CAI. ITT and GST are safe for young patients when performed in experienced centers in a controlled environment.

## 1 Introduction

Adrenal insufficiency is a rare but severe clinical condition and can be life‐threatening [1]. A precise diagnosis is necessary for adequate management. However, the biochemical confirmation of the diagnosis of central adrenal insufficiency (CAI) can be very challenging. In the absence of an extremely low baseline cortisol, cortisol‐stimulating tests (CSTs) are necessary. The insulin tolerance test (ITT) is the gold standard method to diagnose CAI, but there are insecurities concerning the safety and adverse events associated with it, especially in young patients. An ACTH stimulation test can be specific but presents with low sensitivity and methodological problems concerning the ACTH dose [2]. Glucagon stimulation test (GST) results seem to agree with those found in ITT [3], but glucagon can also cause adverse effects. The mechanisms by which glucagon stimulates cortisol secretion are poorly explained. The glycemic fluctuation or the activation of the central noradrenergic pathways possibly stimulates ACTH‐cortisol secretion [4].

Large studies on the biochemical diagnosis of CAI, especially focusing on CST are lacking in the pediatric population, and the literature diverges in determining the cutoff points to diagnose CAI [3, 5, 6]. Moreover, studies show that cutoff values of Fpeak in GST < 496.6 nmol/L can still represent a normal cortisol secretion [7]. Therefore, considering our tertiary center's experience in treating pediatric neuroendocrine diseases, including CAI, we evaluated all pediatric patients who underwent baseline cortisol measurements and cortisol stimulation tests over 15 years to evaluate their safety and accuracy. Although these tests can simultaneously measure cortisol and growth hormone, the present study focuses exclusively on cortisol measurements; growth hormone levels were recorded during testing but were not analyzed.

## 2 Materials and Methods

### 2.1 Study Design

Retrospective cohort study.

#### 2.1.1 Data and Patients

We included all pediatric patients (up to 17 years of age) submitted to cortisol stimulation tests at the University Hospital at Ribeirao Preto Medical School, University of São Paulo, from 2001 to 2016. Medical data was collected and managed using REDCap. Anthropometric data were collected, and standard deviation scores were calculated using the Center for Disease Control curves for patients older than 2 years old, and World Health Organization curves were used for patients younger than 2, with the following formula: SDS = (stature observed−average stature [age and sex]) ÷ Standard deviation (age and sex). The pubertal evaluation was performed through Tanner stages, and patients were considered pubertal with stages equal to or superior to M2 in females and G2 in males. This study was approved by the University Hospital Research Ethics Committee (45517621.1.0000.5440).

No formal sample size calculation was performed. We selected all patients who underwent testing for cortisol and growth hormone deficiency during a 15‐year period in a tertiary referral University Hospital. All patients with suspected hypopituitarism in the northwestern region of São Paulo state are referred to our hospital for care. Therefore, we selected all patients who underwent ITT and GST within a defined time period in the region, thereby characterizing a defined population.

Handling of missing data: The retrospective nature of the study made it impossible to recover data that was not adequately registered in medical files. Therefore, we decided to exclude patients with incomplete files to prevent interference with subsequent analyses. Among 924 children and adolescents who underwent CSTs, 20 (2%) had several missing data points in their medical charts. Since it is a very small sample compared to the study's population, the data were representative. We worked with all the data and not with data imputation.

### 2.2 Cortisol Measurements

Plasma cortisol was determined by the previously described RIA method after extraction [8]. The same method was used throughout the study (2001 to 2016). Samples were measured in duplicates. The minimal detection concentration of cortisol was 33.11 nmol/L, and the intra‐assay and inter‐assay variations were 5% and 10.5%, respectively.

### 2.3 Cortisol Stimulation Tests

ITT and GST were performed at the University Hospital following the same protocols throughout the whole study. Patients and samples were supervised by a physician of the Endocrinology Division. The patients were admitted to the Outpatient Setting with a minimum of an 8‐h fast. For the ITT, the human regular insulin dose was calculated at 0.1 units/kg and administered intravenously at time zero, after samples were collected at this time. Samples of plasma glucose and cortisol were collected at −15 and 0 min for basal measures, and then at 30, 60, 90, and 120 min. ITT was only considered a valid test for the diagnosis of adrenal insufficiency if significant hypoglycemia occurred (nadir < 45 mg/dL or < 50% basal glucose in non‐diabetic patients). During ITT, capillary glucose measurements were performed at −15, 0, 30, 60, 90, and 120 min, or before if the patient presented with signs or symptoms of hypoglycemia. For GST, a 30 mcg/kg dose was calculated (maximum of 1.0 mg) for glucagon, which was administered intramuscularly at time zero, and cortisol samples were collected at times −15 and 0 for basal measures and then 60, 90, 120, 150, and 180 min.

### 2.4 Imaging Studies

Sella turcica and central nervous system Magnetic Resonance Imaging (MRI) were obtained and analyzed at the Imaging Department of the University Hospital. Two experienced endocrinologists evaluated bone age radiographs for this study.

### 2.5 Statistical Analysis

The general characterization of patients was evaluated (frequency and median). The tests' safety was analyzed considering adverse events characterization and frequency. No statistical comparison was made between the number of adverse events in ITT versus GST because of the small number of patients submitted to both tests.

For subsequent analyses, we excluded all patients who did not develop significant hypoglycemia in the ITT and all patients who had previously used glucocorticoids.

According to cortisol concentrations: basal (Fbasal; nmol/L) and peak post‐stimulus (Fpeak; nmol/L), patients were divided into three groups [5, 9, 10]: adrenal insufficiency (Fbasal < 138 nmol/L or Fpeak < 414 nmol/L), indeterminate (Fbasal between 138 and 358.7 nmol/L or Fpeak between 414 and 496.6 nmol/L), and normal (Fbasal > 358.7 nmol/L or Fpeak > 496.6 nmol/L). Cut‐off points were selected based on previously published data and on our University Hospital's protocol. Although 496.6 nmol/L is considered the cutoff for diagnosing adrenal insufficiency in stimulation tests, recent data [7] showed that the indeterminate results interval could represent a normal cortisol response in children. Therefore, we considered it a group that should be analyzed separately.

The proportion of patients allocated in each category according to Fbasal and Fpeak was evaluated in percentages, and the agreement between tests was evaluated by the weighted Kappa coefficient.

ROC curves analyzed cutoff points for Fbasal and Fpeak in the GST and ITT, which were considered the gold standard for calculating sensitivity and specificity. Weighted Kappa was obtained from Fbasal and Fpeak. The ITT and GST agreement was evaluated using the Bland‐Altman method (only selected patients that underwent both tests were analyzed to avoid selection bias). We compared Fbasal and Fpeak in patients with ectopic posterior pituitary and interrupted stalk syndrome and compared them with controls by Student's T‐test. We constructed a Conditional Inference Tree, a non‐parametric class of decision trees, to evaluate possible categorical or numerical variables that could indicate a determinate result in Fpeak in a regression relationship.

We applied a conditional inference tree to model the relationships among all patients’ characteristics, baseline laboratory results, and the reference‐standard outcome, Fpeak. Conditional Inference Trees are a non‐parametric class of decision trees [11]. Both overfitting and variable selection bias inherent to recursive partitioning procedures are mitigated through the use of testing for variable selection and stopping criteria. Conditional inference trees select predictors based on p‐values to determine splits. Therefore, they select variables in an unbiased way. The partitions obtained from conditional inference trees are closer to the true data partition and select the correct covariate more often, making them more appropriate for diagnostic purposes. This approach was chosen because it can capture non‐linear effects and higher‐order interactions without requiring pre‐specification. The resulting tree structure provides an interpretable framework for identifying subgroups with differing disease probabilities. We analyzed all variables that could interfere with the test result (age, neonatal signs and symptoms of hypopituitarism, exposure to radiotherapy, height SDS, baseline laboratory tests (IGF1, free T4, fasting glucose, basal cortisol, sodium, potassium, anatomical hypothalamic‐pituitary and other CNS abnormalities, and previous diagnostic hypothesis of CAI). In the conditional inference trees, we wanted to evaluate variables and the prediction of Fpeak, but not suggest different cutoffs for ITT or GST. Therefore, we did not conduct sensitivity analyses. The software R version 4.0.3 was used.

## 3 Results

A total of 924 cortisol stimulation tests were carried out. Among these patients, 20 were excluded due to a lack of data or duplicate records. After excluding duplicates or patients with insufficient data in their medical files, 904 patients were analyzed. The ITT (67%) was the most frequent test, followed by the GST (33%).

Most patients were male (60.1%), prepubertal at admission (70.4%), with a median age of 10.1 (0.1−17.1) years. Suspicion of CAI motivated the performance of the test in 21.9%, while most patients had GH and cortisol secretion evaluated for short stature (76.8%). One quarter (24.5%) had signs of delayed cognitive or motor development, and one‐third (31.4%) had dysmorphic features at examination (mostly associated with Turner and Silver‐Russell syndromes, with the former confirmed by karyotype in 61 patients). Reported previous neonatal signs and symptoms of hypopituitarism were found in 17.2% of the patients. They included seizures, hypoglycemia, cryptorchidism, micropenis, and prolonged jaundice. At admission, some patients were receiving prescriptions for glucocorticoids (5.5% oral, 2.9% inhaled, and 1.8% topical), taking anticonvulsant medications (8.4%), and undergoing either chemotherapy (2.3%) or radiotherapy (2.9%) for cancer. Among the 6.4% of patients who underwent molecular analysis, one patient had a *PIT1* mutation, one had an *SHH* mutation, and two had mutations in the *GH* gene. In one patient, Prader‐Willi syndrome was confirmed.

MRI was performed in 55% of the patients. The most common findings included ectopic neurohypophysis (in 67 patients), hypoplastic anterior pituitary (in 71 patients), and hypoplastic or absent pituitary stalk (in 33 patients). Some patients had other classic malformations associated with hypopituitarism and septo‐optic dysplasia, such as agenesis of corpus callosum (24 patients), optic nerve alterations (18 patients), and agenesis of septum pellucidum (14 patients).

More than half of the ITTs performed resulted in effective hypoglycemia (59.1% < 45 mg/dL and 50.4% < 50% basal glycemia), confirming the adequacy of the test. In 40.5% of the patients, mild hypoglycemia symptoms, such as hunger, tremors, sweating, nausea, and somnolence, were reported. However, no patients presented seizures or needed assisted ventilation, and no patients presented with permanent sequelae. Among all 621 patients who underwent ITT, only nine remained in observation at the outpatient setting for a few hours after the test, but none needed hospitalization. The median time of hypoglycemia recovery was 30 min, and some patients received oral (8.5%) or intravenous glucose (10.1%).

In the GST, most of the adverse effects reported were associated with gastrointestinal symptoms, such as nausea (4.2%) and vomiting (4.2%). Fewer patients reported somnolence (2.1%). Four patients (1.6%) needed additional observation in the outpatient setting, but none required hospitalization.

Among the 904 evaluated patients, 1.9% presented with complete anterior and posterior hypopituitarism, 2.7% presented with complete anterior hypopituitarism, 11.4% partial hypopituitarism, and 1.2% presented with isolated CAI. The most affected axis in the patients with partial anterior hypopituitarism was GH (81%), followed by TSH (61%) and ACTH (48%). Disturbances in the gonadal axis were confirmed in 39% of patients.

**Figure 1:**
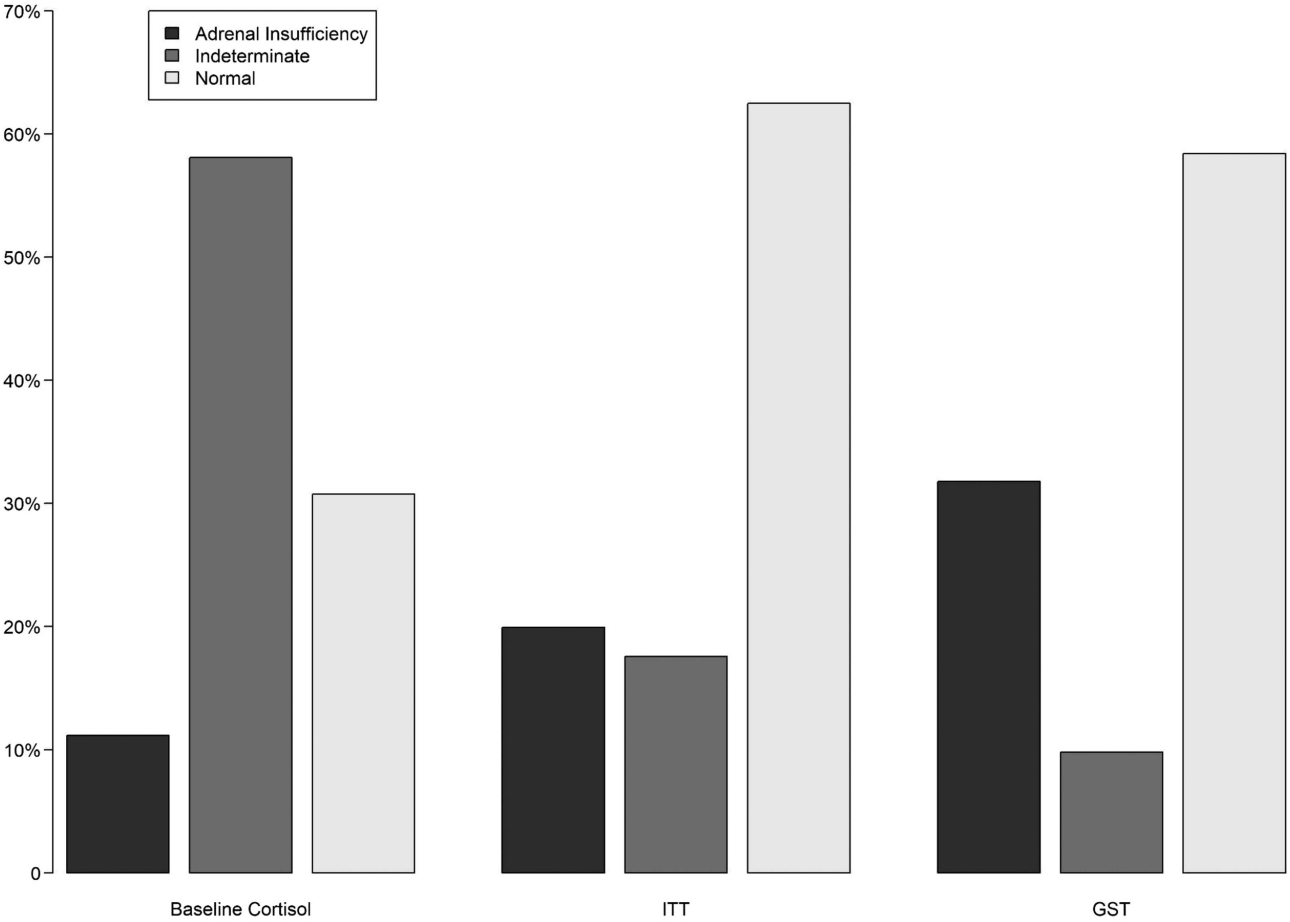
Comparison of diagnostic classifications across cortisol assessment methods. Proportion (%) of patients categorized as Adrenal Insufficiency, Indeterminate, or Normal based on Basal Cortisol (Total *n* = 805), Insulin Tolerance Test (ITT) (Total *n* = 489), and Glucagon Stimulation Test (GST) (Total *n* = 344). For Baseline Cortisol, the majority of patients were classified as Indeterminate. Agreement between diagnostic methods was assessed using weighted kappa to account for the ordinal nature of the diagnostic categories: 0.25 (CI 95%: 0.2−0.3). Baseline cortisol shows a higher tendency to classify patients as Indeterminate compared to dynamic stimulation tests.

Figure 1 shows the proportion of patients allocated in each category for Fbasal and Fpeak results. The weighted Kappa coefficient was 0.25 (CI 95%: 0.2−0.3), demonstrating a weak agreement between basal cortisol and CST in the allocation of patients among categories.

**Figure 2:**
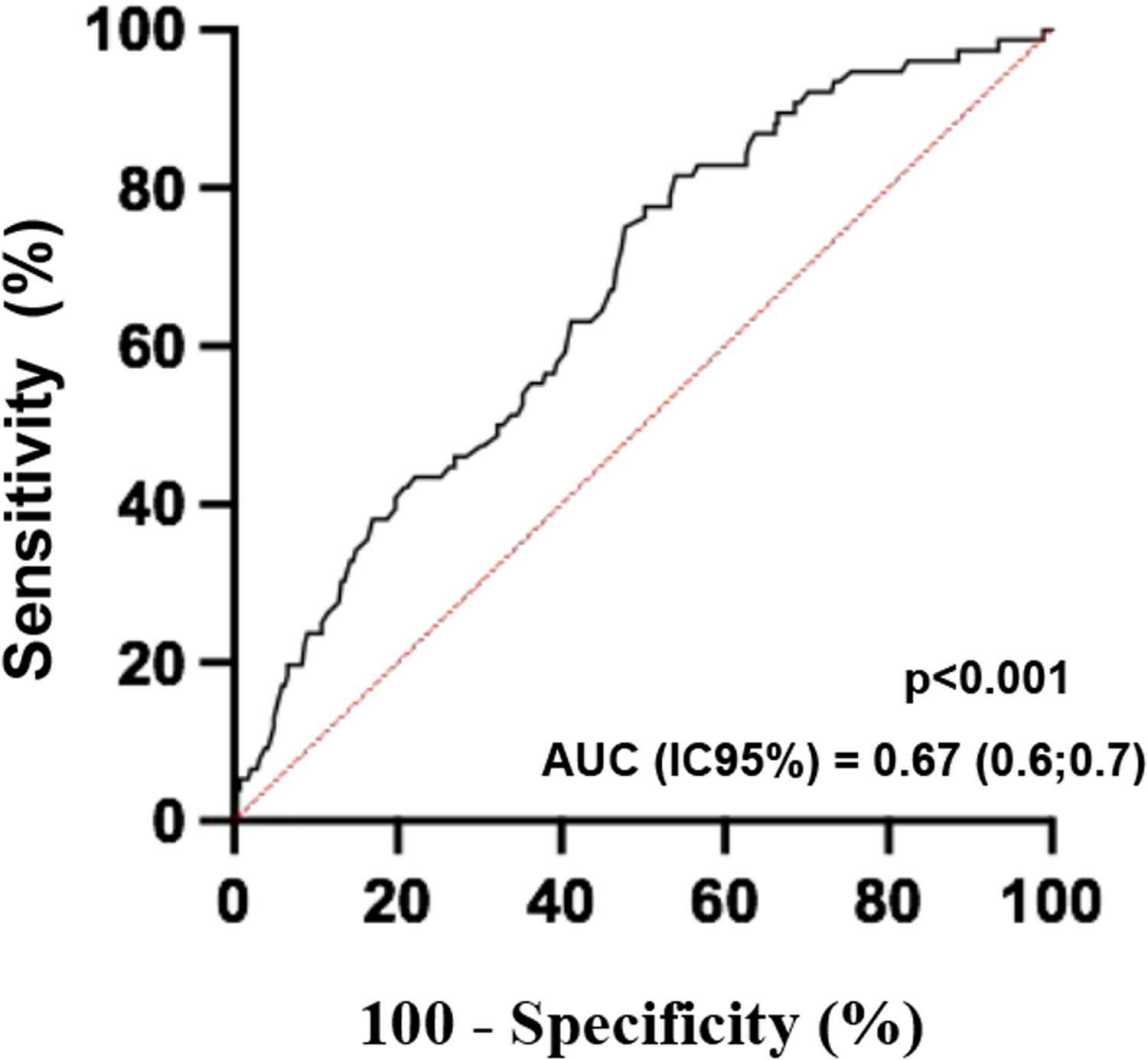
Receiver Operating Characteristic (ROC) curve analysis of baseline cortisol. Performance of baseline cortisol in predicting central adrenal insufficiency in a pediatric cohort. *n* = 365. The area under the curve (AUC) is 0.67 (95% Confidence Interval: 0.60–0.70). *p* < 0.001. The Y‐axis represents Sensitivity (%) and the X‐axis represents 100—Specificity (%).

ROC curves evaluating the sensitivity and specificity of baseline cortisol (Figure 2) resulted in an area under the curve of 0.67. We observed that the cut‐off value of 96.6 nmol/L had the highest diagnostic accuracy (LR+ of 7.6), with a high specificity (99%) despite a low sensitivity (5.3%). The cut‐off of 138 nmol/L had lower diagnostic accuracy (LR+ 2.2), maintaining low sensitivity (9.2%) but with high specificity (96%). The cut‐off value of 358.7 nmol/L showed a considerable increase in sensitivity (82%) but with low specificity. The CSTs did not add diagnostic value in patients with Fbasal results over 358.7 nmol/L, and these patients did not present with CNS abnormalities or pathologies that increased risk for CAI. The ROC analysis yielded an AUC of 0.67, indicating poor diagnostic performance and limited ability to reliably distinguish between diagnostic groups.

**Figure 3:**
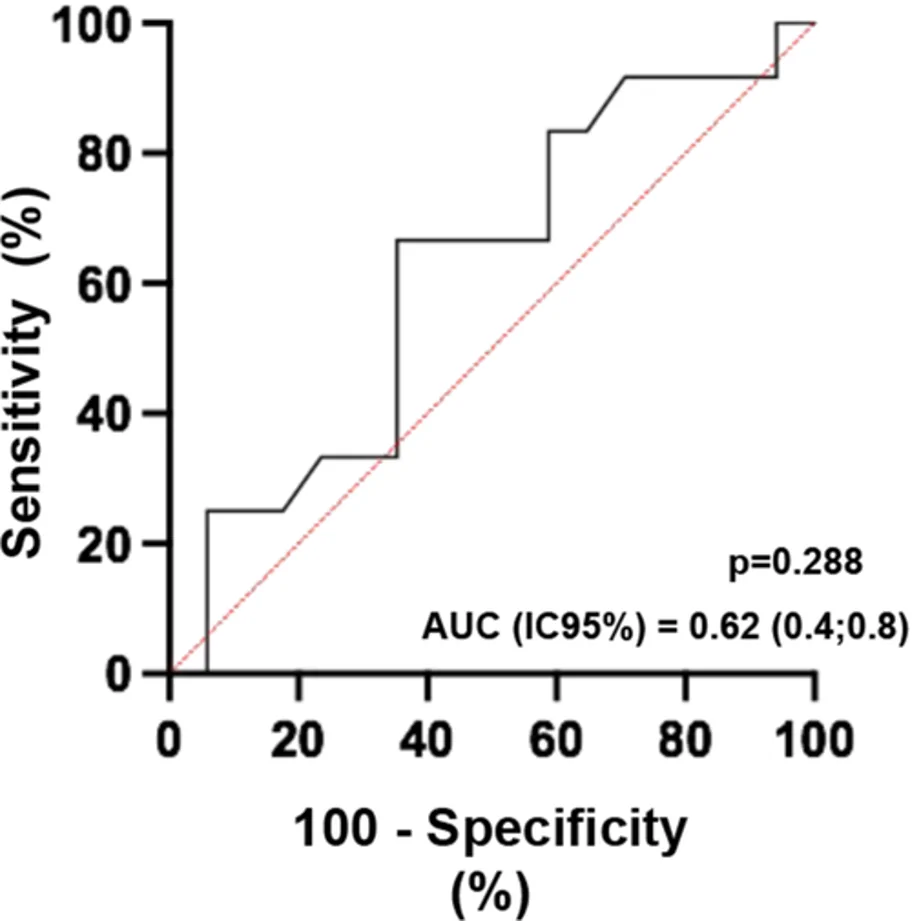
Receiver Operating Characteristic (ROC) curve analysis of the Glucagon Stimulation Test (GST). Diagnostic performance of peak cortisol levels during the GST for identifying central adrenal insufficiency in a pediatric cohort. *n* = 49 children. The area under the curve (AUC) is 0.62 with a 95%Confidence Interval (IC 95%) of 0.40–0.80. *p* = 0.288. The vertical axis indicates Sensitivity (%) and the horizontal axis indicates 100—Specificity (%).

For diagnosing CAI using Fpeak GST, we observed in the ROC curve (Figure 3) that a cutoff point of 303.5 nmol/L had the highest diagnostic accuracy (LR+ of 2.8), a good specificity of 94%, but low sensitivity (17%). The observed LR+ values (e.g., 2.8) indicate weak diagnostic evidence, suggesting only a small increase in post‐test probability. The cutoff of 496.6 nmol/L showed low sensitivity (42%) and specificity (65%). However, the area under the curve was 0.62, and the number of patients who underwent both GST and ITT was only 49. There was a small sample size for GST ROC analysis, which underpowers this analysis, and results should be cautiously interpreted and not implicated for clinical decisions.

**Figure 4:**
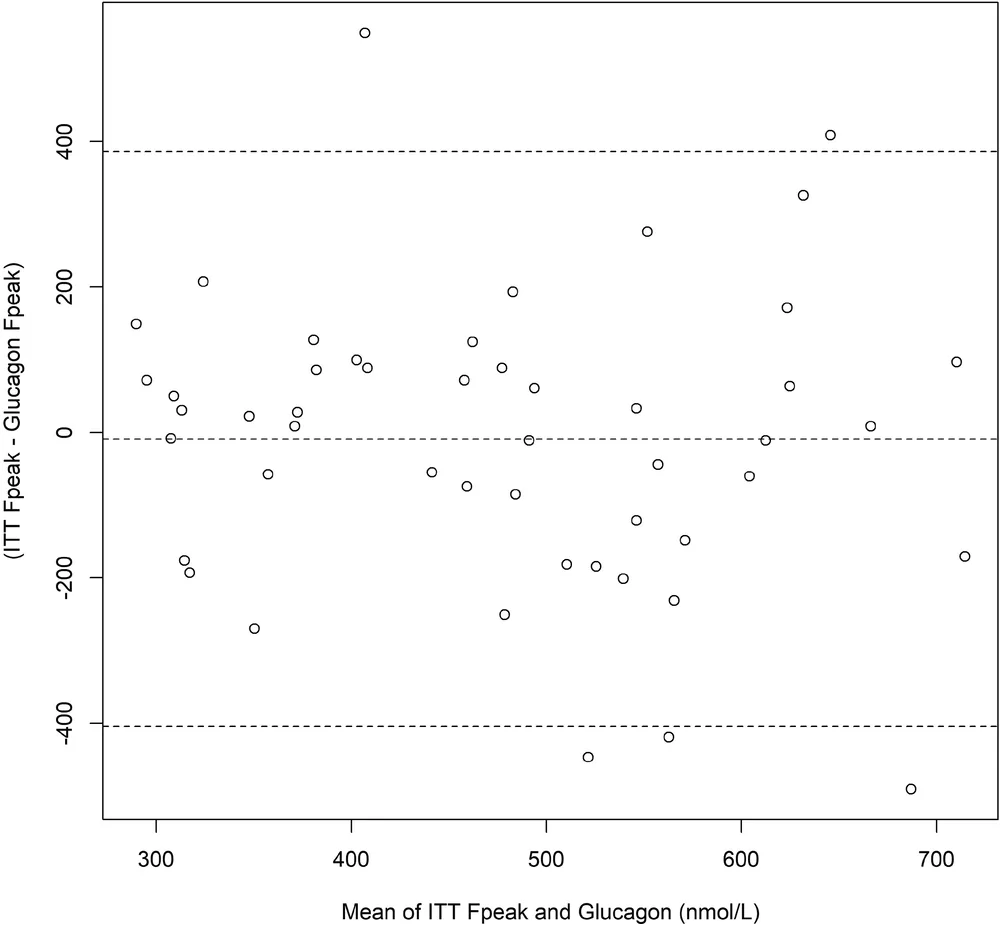
Bland‐Altman plot assessing agreement between peak cortisol levels. Comparison between peak cortisol concentrations reached during the Insulin Tolerance Test (ITT) and the Glucagon Stimulation Test (GST). *n* = 49. The Y‐axis represents the difference between ITT peak and GST peak (ITT‐GST Fpeak). The X‐axis represents the mean of the two peak values. The horizontal dashed lines represent the Mean Difference and the 95% Limits of Agreement.

When we analyzed the results of the 49 patients who underwent both CST, ITT, and GST, we observed a good agreement between results (Figure 4), especially when considering results lower than 496.6 nmol/L for Fpeak, which are diagnostic of CAI.

**Figure 5:**
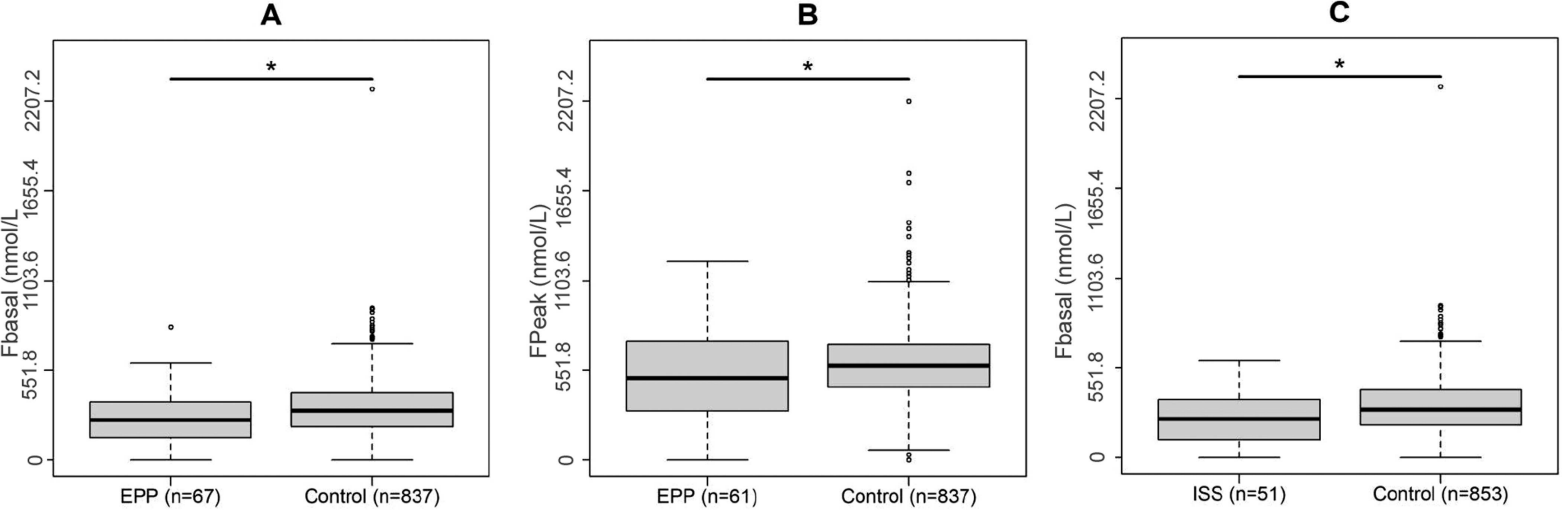
Comparison of cortisol levels between pituitary structural abnormality groups and controls. (A) Baseline Cortisol (Fbasal): Patients with ectopic posterior pituitary (EPP, *n* = 67) versus controls (*n* = 837). (B) Peak Cortisol (Fpeak): Patients with EPP (*n* = 61) versus controls (*n* = 837). (C) Baseline Cortisol (Fbasal): Patients with interrupted stalk syndrome (ISS, *n* = 51) versus controls (*n* = 853). Units: All values are expressed in nmol/L. Test: Groups were compared using the Student's *T*‐test. *p < 0.01 indicates a statistically significant difference between the patient group and the control group. Boxplots represent the median and interquartile range (IQR); whiskers represent 1.5 times IQR, and open circles represent outliers.

Subgroups were analyzed to compare Fbasal and Fpeak in patients with pituitary anatomical malformations. Patients with ectopic posterior pituitary presented with significantly lower Fbasal and Fpeak (Figures 5a,b, respectively). Fbasal was also lower in patients with interrupted stalk syndrome (Figure 5c), but no difference was observed in Fpeak.

**Figure 6:**
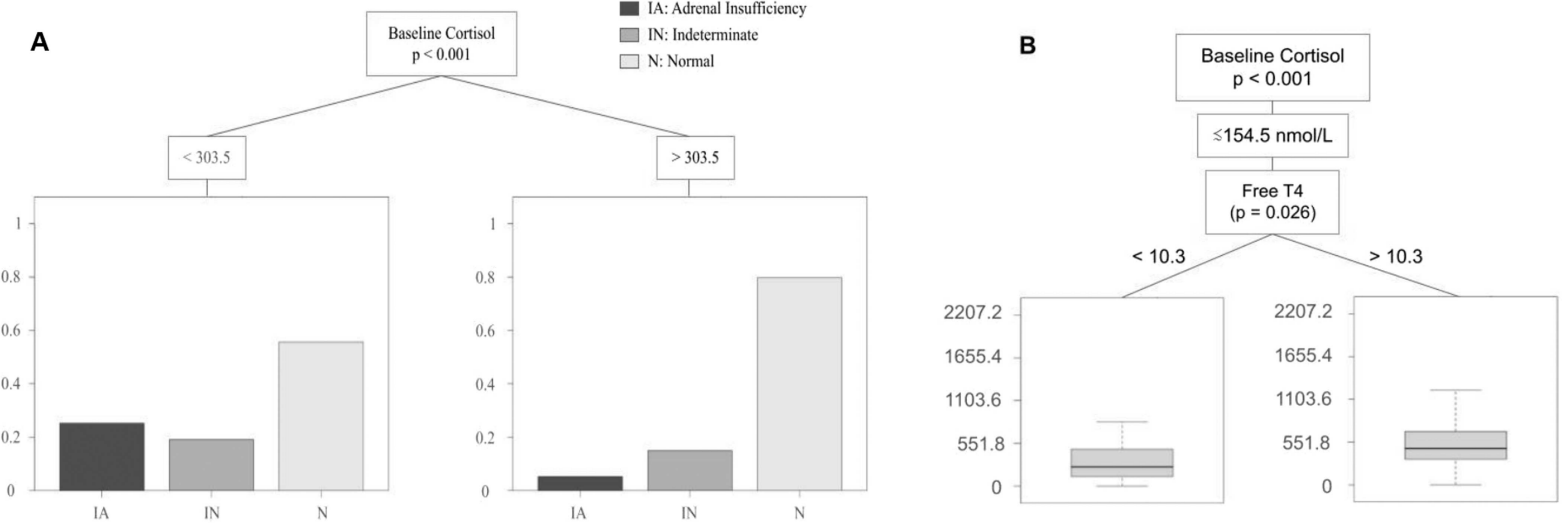
Conditional inference tree analysis for the prediction of stimulated cortisol response. Decision tree models demonstrating the association between baseline variables and peak cortisol levels (F_peak) during stimulation testing. (A) Baseline Cortisol Model: Classification of F_peak based on baseline cortisol cutoff points (e.g., < 303.5 vs. > 303.5 nmol/L). (B) Multivariate Model: Analysis showing that F_peak is significantly associated with baseline cortisol ≤ 154.5 nmol/L (*p* < 0.001) and Free T4 levels (*p* = 0.026). A Free T4 level < 10.3 pmol/L further identifies a subgroup with lower stimulated cortisol. Units: Cortisol values are presented in nmol/L. Free T4 is presented in pmol/L. A conditional inference tree algorithm was used to partition the data based on significance (*p* values are shown at each node). Terminal Nodes: The boxplots at the bottom represent the distribution of Fpeak (nmol/L) for each identified subgroup. Boxplot Representation: The center line represents the median; the box represents the interquartile range (IQR); and whiskers represent 1.5 times IQR.

We evaluated which variables were associated with cortisol‐stimulated responses using multiple regression analysis. These results are shown in Figures 6a,b, conditional inference trees. Baseline cortisol and free T4 were the only variables that could differentiate between groups (*p* < 0.001). A cutoff of 303.49 nmol/L for baseline cortisol was able to correctly classify most individuals between normal and having CAI (Figure 6a). However, 20% of patients with baseline cortisol greater than 303.5 nmol/L still presented with cortisol peak levels in the stimulation tests under 496.6 nmol/L. Lastly, baseline cortisol lower than 154.5 nmol/L and a free T4 lower than 10.3 pmol/L were associated with a peak in cortisol stimulation test under 414 nmol/L (Figure 6b, *p* < 0.001 and *p* = 0.026), a result compatible with CAI diagnosis.

## 4 Discussion

In this report, we analyzed a large cohort of children submitted to the same protocol of CST and cortisol assay to evaluate the diagnostic accuracy of ITT and GST to differentiate children with CAI from those with an intact HPA axis.

After evaluating the CST's safety, we observed only minor adverse events, and no major events occurred. Most episodes of hypoglycemia were mild, and all were solved with glucose supplementation. We found similar results for GST. The most frequent side effects were vomiting and nausea, and most resolved spontaneously. As previously described by Hanukoglu and Weisglass [12], most pediatric endocrinologists fear ITT and its complications and avoid submitting pediatric patients to definitive and conclusive CST. Retrospective studies might lead to underestimation of adverse events due to inaccurate documentation in medical records; however, the protocols of our Endocrinology Department are very strict for charting, and patients with incomplete data were excluded from the study, as mentioned previously. Patients at higher risk of complications of hypoglycemia were not submitted to ITT, thereby limiting the generalizability of the safety findings. Nevertheless, patients with contraindications to ITT were tested with GST, as recommended in current guidelines, and experienced very few adverse effects.

Baseline cortisol had low accuracy for diagnosing CAI at most cutoff points and classified most patients as indeterminate when compared to stimulation tests. We found that a baseline cortisol cutoff of 96.6 nmol/L had the highest specificity to diagnose CAI, but with low sensitivity, which was similar to previous results [5, 10]. Cutoff points higher than 303.5 to 358.7 nmol/L had an improved sensitivity, but at the expense of a significant decrease in specificity. These findings show that baseline cortisol may confirm CAI diagnosis at very low levels, but presents low accuracy at higher concentrations. This fact highlights the need to perform CSTs in most pediatric patients at risk of CAI.

Two variables were associated with lower Fpeak when associated with low Fbasal: central hypothyroidism and ectopic posterior pituitary. Therefore, the diagnosis of CAI should be strongly considered in patients with low Fbasal (<154.5 nmol/L) in the presence of central hypothyroidism and ectopic posterior pituitary.

Patients with contraindications for ITT should be submitted to other stimuli for cortisol secretion, such as glucagon or synthetic ACTH_11‐24_. We observed a good agreement between the results of GST and ITT. This finding is consistent with previous data [3] and is more evident at cortisol concentrations lower than 496.6 nmol/L, which is usually considered diagnostic for CAI. For GST, the 303.5 nmol/L Fpeak cutoff point had the highest diagnostic accuracy to identify patients with CAI. This finding is in agreement with those in the literature suggesting lower cutoff points for the diagnosis of CAI with higher specificity [3, 7].

Overall, the synthetic ACTH11‐24 stimulation test is more commonly used than the ITT in pediatric practice, largely because of safety concerns about hypoglycemia. Although it is not routinely used in our setting for diagnosing CAI, it remains a widely accepted and guideline‐recommended test. The ACTH_11−24_ test has high specificity and is therefore useful for confirming secondary adrenal insufficiency; however, its sensitivity is limited, particularly in cases of recent‐onset CAI, where adrenal atrophy may not yet be present due to insufficient duration of endogenous ACTH deficiency [2, 5, 13]. Consequently, false‐negative results may occur in this context [5, 13]. In addition, the standard 250 µg dose may be supraphysiologic, while the low‐dose (1 µg) test may be subject to methodological variability. A meta‐analysis has reported low sensitivity (36%−69%) but high specificity (91%–99%) for this test, and similar accuracy in the standard and the low‐dose tests [2]. Therefore, both baseline cortisol and ACTH_11–24_ stimulation tests may be useful for confirming the diagnosis, but are less reliable for excluding CAI, particularly in early disease.

The lack of an objective evaluation of responsiveness to glucagon limits the interpretation of the positive results, and this could explain the higher number of CAI diagnoses in GST compared to ITT.

The most significant limitation of this study is its retrospective nature. However, we evaluated a large number of patients under the same protocol from a single tertiary referral center. Moreover, we applied the same cortisol assay during the 15 years of the study in all patients. The diagnosis of CAI is challenging but still extremely important in order to treat a severe disease and to avoid complications of unnecessary treatment with glucocorticoids in misdiagnosed patients. This study confirms that ITT and GST appear safe when performed in experienced centers in a controlled environment, and that most adverse events associated with these tests were mild. Moreover, it shows that baseline cortisol is insufficient to diagnose CAI in most patients. However, in the presence of central hypothyroidism and ectopic posterior pituitary, lower Fbasal predicts lower Fpeak, strongly suggesting the diagnosis of CAI.

## 5 Conclusions

Fbasal does not present good diagnostic accuracy at concentrations higher than 96.6 nmol/L in children and adolescents with clinical suspicion of CAI. Low Fbasal (< 154.5 nmol/L) can predict lower Fpeak in the patient also presenting central hypothyroidism and ectopic posterior pituitary. We observed good agreement between Fpeak results below 496.6 nmol/L in ITT and GST. A Fpeak cutoff of 303.5 nmol/L in GST suggested only a small increase in post‐test probability. Our data show that ITT and GST appear safe in young patients. We observed good agreement between Fpeak results below 496.6 nmol/L in ITT and GST. A Fpeak cutoff of 303.5 nmol/L in GST suggested only a small increase in post‐test probability.

## Conflicts of Interest

The authors declare no conflicts of interest.

## Data Availability

The data that support the findings of this study are available on request from the corresponding author. The data are not publicly available due to privacy or ethical restrictions.
